# Optimization of Friction Stir Spot Welding Process Using Bonding Criterion and Artificial Neural Network

**DOI:** 10.3390/ma16103757

**Published:** 2023-05-16

**Authors:** Deok Sang Jo, Parviz Kahhal, Ji Hoon Kim

**Affiliations:** 1 School of Mechanical Engineering, Pusan National University, Geumjeong-gu, Busan 46241, Republic of Korea; jods90@pusan.ac.kr (D.S.J.); pkahhal@gmail.com (P.K.); 2 Hybrid Innovative Manufacturing & Engineering Center, Pusan National University, Geumjeong-gu, Busan 46241, Republic of Korea; 3 Department of Mechanical Engineering, Ayatollah Boroujerdi University, Boroujerd 69199-69737, Iran

**Keywords:** friction stir spot welding, bonding criterion, coupled eulerian–lagrangian, artificial neural network

## Abstract

The objectives of this study were to analyze the bonding criteria for friction stir spot welding (FSSW) using a finite element analysis (FEA) and to determine the optimal process parameters using artificial neural networks. Pressure-time and pressure-time-flow criteria are the bonding criteria used to confirm the degree of bonding in solid-state bonding processes such as porthole die extrusion and roll bonding. The FEA of the FSSW process was performed with ABAQUS-3D Explicit, with the results applied to the bonding criteria. Additionally, the coupled Eulerian–Lagrangian method used for large deformations was applied to deal with severe mesh distortions. Of the two criteria, the pressure-time-flow criterion was found to be more suitable for the FSSW process. Using artificial neural networks with the bonding criteria results, process parameters were optimized for weld zone hardness and bonding strength. Among the three process parameters used, tool rotational speed was found to have the largest effect on bonding strength and hardness. Experimental results were obtained using the process parameters, and these results were compared to the predicted results and verified. The experimental value for bonding strength was 4.0 kN and the predicted value of 4.147 kN, resulting in an error of 3.675%. For hardness, the experimental value was 62 Hv, the predicted value was 60.018 Hv, and the error was 3.197%.

## 1. Introduction

**Figure 1:**
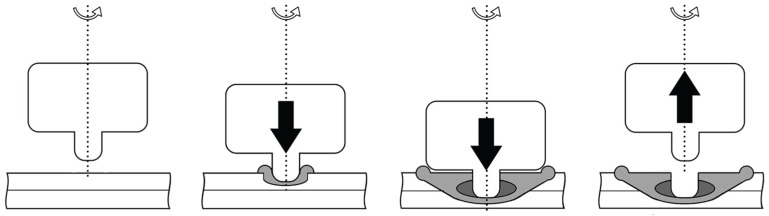
Schematic of the friction stir spot welding (FSSW) procedure.

Recently, aluminum alloys have been widely used in industries using sheet materials to increase fuel efficiency and improve impact resistance. Most research has focused on applying aluminum alloys to tailored blanks, such as tailor-welded and tailor-rolled blanks [1]. However, due to their high thermal conductivity and low melting point, aluminum alloys have low weldability in resistance spot welding and laser welding, making them difficult to process into patchwork blanks [2,3]. Therefore, friction stir spot welding (FSSW) is often used instead of conventional welding methods. FSSW is a convenient solid-state welding process for welding aluminum alloys [4]. Compared to conventional welding techniques, FSSW has various advantages, such as low cost and eco-friendliness. Figure 1 shows a schematic of the FSSW process. Typically, the welding process involves plunging a rotating tool into the specimen, followed by stirring; the tool is retracted after the welding is complete.

Nevertheless, numerical simulation of the FSSW process is problematic due to mesh distortions in the finite element analysis (FEA). Accordingly, in the FEA of the FSSW process, the arbitrary Lagrangian–Eulerian (ALE) and coupled Eulerian–Lagrangian (CEL) methods are often used to account for large deformations. Xiong et al. developed a numerical model for AA2524-T3 refill FSSW using the CEL method to investigate the correlations between the process parameters, and found a relationship exists between the grain size and tool rotational speed [5]. Chu et al. studied the joint formation and defect evolution in probeless FSSW using a three-dimensional CEL model. According to the FEA results, the highest temperature and plastic strain were located at the inner edge of the shoulder, owing to the higher heat flux around the shoulder [6]. El-Sayed et al. reviewed different techniques for friction stir welding (FSW) and assessed the current state of finite element modeling using the ALE method [7].

However, it is difficult to determine the effectiveness of the FSSW process based solely on an FEA. Therefore, bonding is often verified and confirmed based on the bonding criterion used in the solid state. Some studies have analyzed solid bonding mechanisms in processes such as roll bonding and porthole die extrusion while employing appropriate bonding criteria. Donati and Tomesani investigated the impacts of die design on the product quality in aluminum profiles extruded with seam welds [8]. Donati et al. analyzed the process parameters and distinct die geometries utilized in the extrusion of a hollow profile featuring a seam weld [9]. Through a numerical analysis of the extrusion processes, they obtained local conditions of the experimentally produced welds and developed a bonding criterion applicable to various welding conditions. Moreover, Donati et al. found that using a larger welding chamber could significantly improve product deformability [10]. Gianluca Buffa et al. conducted experimental tests, a microstructural analysis, and an FEA to evaluate the reliability and effectiveness of using bonding criteria previously developed for other processes for FSW [11]. To implement the considered criteria and understand the material flows during FSW, Buffa et al. employed a validated numerical model to compute the essential field parameters and found that the pressure-time-flow criterion was a more suitable bonding criterion than the pressure-time criterion in the FSW process [12]. The process parameter optimization was performed using an artificial neural network (ANN). Nejad et al. investigated the fatigue crack growth rate and fracture behaviors of an aluminum 2024-T351 alloy and employed an adaptable optimization algorithm to determine the optimal welding parameters. They also conducted a sensitivity analysis to evaluate the impacts of the rotational and feed rates on the fatigue crack growth rate and fracture toughness [13]. Kahhal et al. combined a response-surface methodology and multi-objective particle swarm optimization algorithm to investigate the multi-objective optimization of mechanical properties during the FSW of an AH12 1050 aluminum alloy [14]. Ramesh studied the role of the process parameters in the FSW processes [15]. In earlier studies that relied solely on FEA, it was challenging to identify the materials suitable for FSSW, along with their corresponding bonding strength and hardness. However, by incorporating the FEA results into the bonding criteria, it is now possible to determine the feasibility of bonding and predict the weldability without conducting experiments. In this study, an FEA is performed using the CEL technique of the ABAQUS commercial software, which deals with the severe mesh distortions in the FEA of the FSSW process. The results from the FEA analysis were applied to two solid-state bonding criteria to identify the most suitable bonding criteria for the FSSW process. In addition, the optimal process parameters were derived using an ANN technique with hardness and bonding strength as the objective functions. The reliability of this approach was confirmed by comparing the predicted and experimental results.

## 2. Methods

### 2.1. Experiments

**Figure 2:**
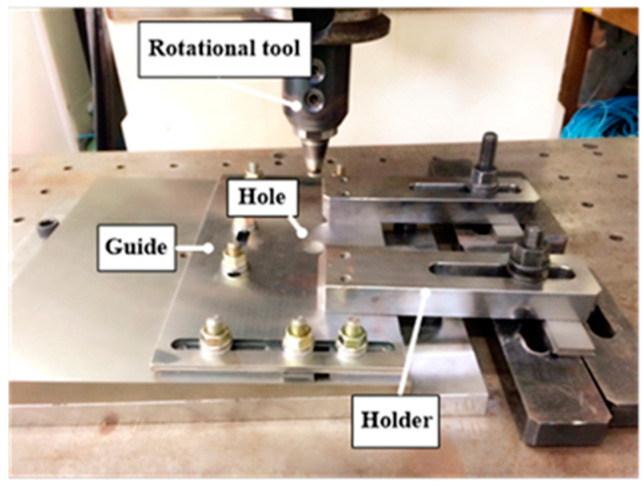
FSSW fixture.

**Figure 3:**
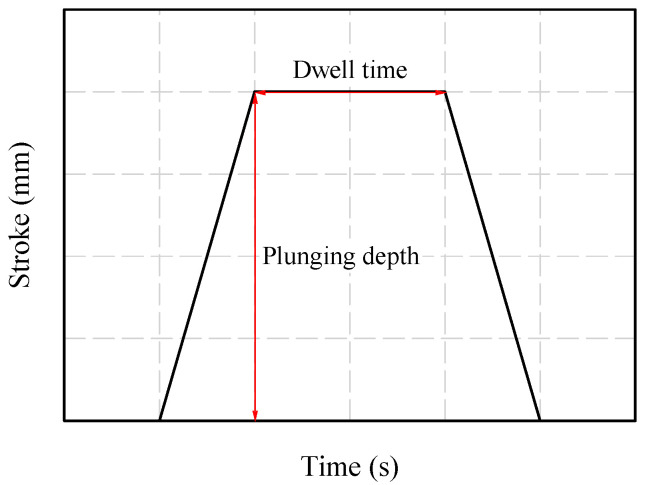
Stroke profile in the FSSW process.

**Figure 4:**
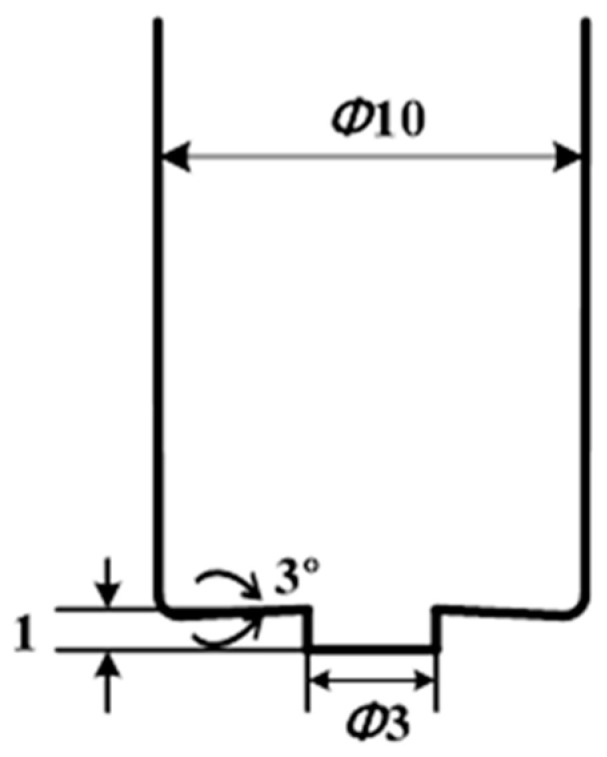
FSSW tool geometry.

**Figure 5:**
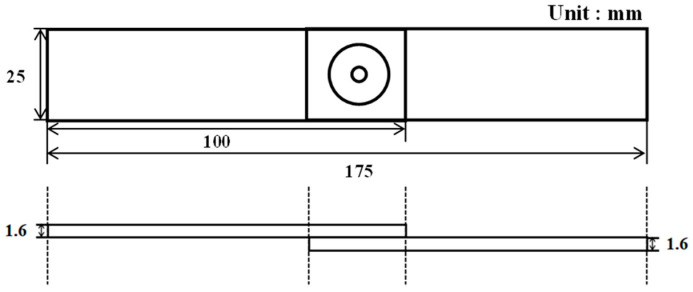
Typical specimen for lap-shear test.

A 1.6-mm-thick sheet of AA6061-T6 was used for the experiment. Table 1 lists the mechanical properties of AA6061-T6 [16]. FSSW was conducted on a WINGEN friction stir machine with a 20-kW spindle motor. As shown in Figure 2, the specimen was fixed and the experiment was performed by changing the process parameters. During the FSSW process, various process parameters can affect the welding quality. FSSW process parameters include tool rotation speed (RS), dwell time (DT), plunging depth (PD), tilting angle, and pin geometry. Figure 3 shows a graph of stroke length over time in the FSSW process; the maximum stroke is the PD, and DT is the duration at the maximum stroke. The geometry of the rotating tool (including the size and configuration of its pin and surface area in contact with the shoulder region) can impact both the heat generated through friction and the resulting deformation behavior. The friction behavior between the sheets and FSSW tool is significantly influenced by the height of the pin and area of the shoulder region in the tool geometry. In the FSSW process, the region of the shoulder in contact with the material is a primary source of heat generation. The pin in the FSSW process is responsible for the intense stirring and serves as a significant source of heat generation within the weldment. The PD in the FSSW process can impact both the level of pressure exerted between the tool surface and sheets and the amount of generated heat. RS and DT can also significantly impact the welding quality, including weldment microstructures and bonding strength. As a result, the quality of welding, including strength, ductility, and characteristics of the heat-affected zone, is determined by both the tool geometry and the process parameters. Among the various process parameters with the strongest influences on welding quality and easily changeable by users, RS, DT, and PD were selected in this study. Process parameter sets for the test cases are shown in Table 2. A total of 81 specimens were manufactured (three for each case), with the average value of the parameters considered. Figure 4 shows the tool used in the experiment. A lap-shear test, prepared according to ASTM D3163 and performed using a universal testing machine (Manufacturer: R&B, Daejeon, Republic of Korea) at a loading rate of 1.3 mm/min, was conducted on a test specimen manufactured using FSSW (Figure 5). The lap-shear test was used to determine the bonding characteristics and bonding strength [4,16].

### 2.2. Finite Element Analysis (FEA)

FEA was utilized to examine the outcomes of the FSSW process. The simulation used the commercial software ABAQUS/2021 and a workstation equipped with E5-2640 CPU and 64.0 GB RAM. FEA was performed using the CEL method supported by the ABAQUS software, as mesh distortions do not occur under large deformations during FSSW.

The FEA implemented boundary conditions and plunge speeds utilized in the actual experiments and used Eulerian parts with EC3D8RT temperature-displacement elements to represent the workpieces, whereas the tool was modeled as a rigid Lagrangian body with the same diameter as the actual part. The workpiece measured 25 × 25 mm and had a thickness of 3.2 mm, with a 1.0-mm void layer at the top. The workpiece was partitioned into two equal-thickness sub-regions—upper and bottom sheets. Subsequently, material properties were assigned to each worksheet using the volume fraction technique. The FEA made several assumptions: the tool was represented as a rigid body, the workpieces followed the temperature- and strain rate-dependent elastic-plastic Johnson–Cook model, the initial temperature of the workpiece tool was 22 °C, and the tool was assumed to be isothermal. Equation (1) presents the utilization of the Johnson–Cook model for representing the plasticity of the material, while Equation (2) shows the adoption of the Johnson–Cook damage model. The model parameters used for AA6061 were based on previous references [6,16,17,18]. The equations are defined as:

(1) $$\sigma =(A+B\varepsilon^{n})(1+Cln(\dot{\varepsilon }/{\dot{\varepsilon }}_{0}))[1-{\left((T-T_{r})/(T_{m}-T_{r})\right)}^{m}]$$

and

(2) $${\overset{-}{\varepsilon }}_{f}=\left[D_{1}+D_{2}e^{D_{3}\sigma *}\right]\left[1+D_{4}\varepsilon *\right][1+D_{5}T*].$$

The melting temperature equation involves the constants A,B,C,n, and m specific to the material as well as the ambient temperature $T_{r}$, plastic strain ε, and plastic strain rate $\dot{\varepsilon }$. The model includes the material-specific parameters $D_{1}$, $D_{2}$, $D_{3},D_{4}$, and $D_{5}$ as well as the triaxiality ratio σ*, dimensionless strain rate ε* (representing the ratio of the viscoplastic strain rate to a reference strain rate), and homologous temperature of the material T*.

**Figure 6:**
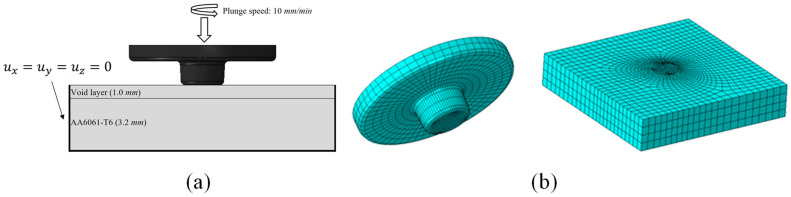
Schematic finite element (FE) model: (**a**) Boundary conditions of the FE model (**b**) Mesh of FE model.

The following boundary conditions were applied in the FEA: the tool was allowed to translate and rotate only in the Y-direction, with the workpieces fixed in the X-direction. Figure 6a shows the boundary conditions of the FEA model and Figure 6b shows the mesh of the FEA model. The model incorporated the temperature-varying thermal properties of the AA6061-T6 sheets, including their thermal conductivity and specific heat, as shown in Table 3.

The main source of heat in the FSSW process is the friction between the workpiece and rotating tool, with Coulomb’s friction law utilized to describe their contact. The specified friction coefficients are listed in Table 3.

### 2.3. Bonding Criterion

As mentioned in Introduction, two criteria are used for solid-state bonding. The first criterion is the pressure-time criterion W [21], based on the integral in time of the ratio between the material contact pressure and flow stress; W is calculated as follows:

(3) $$W=\int_{0}^{t}\frac{p}{\sigma }dt≅\sum_{j}\frac{p_{j}}{\sigma j}∆t_{j}\;.$$

The flow stress of the material at a given temperature, strain, and strain rate is represented by σ and the contact pressure at the interface is denoted by p. A threshold value from a previous study was used in this study [22]. The second criterion is the pressure-time-flow criterion $W^{\prime }$ [7], calculated as follows:

(4) $$W^{\prime }=\int_{0}^{t}\frac{p}{\sigma }vdt≅\sum_{j}\frac{p_{j}}{\sigma j}v_{j}∆t_{j}\;.$$

In the above equation, v is the velocity of the specified node. W has a time unit (s), whereas $W^{\prime }$ has a length unit (mm). $W^{\prime }$ can be considered the “equivalent length” of material flow. In other words, when a particle of a substance reaches a suitable temperature and pressure, it must have a certain minimum velocity for an appropriate amount of time.

Experiments were conducted according to the specifications provided in Table 2 to evaluate the suitability of the considered bonding criteria for FSSW. Lap-shear tests were conducted to check the bonding according to each process parameter and measure the bonding strength. In addition, to identify the bonding criterion suitable for the FSSW process among the two-bonding criterion, the values of W and $W^{\prime }$ were derived by substituting the results of the FEA into the two bonding criteria.

### 2.4. Artificial Neural Network (ANN)

**Figure 7:**
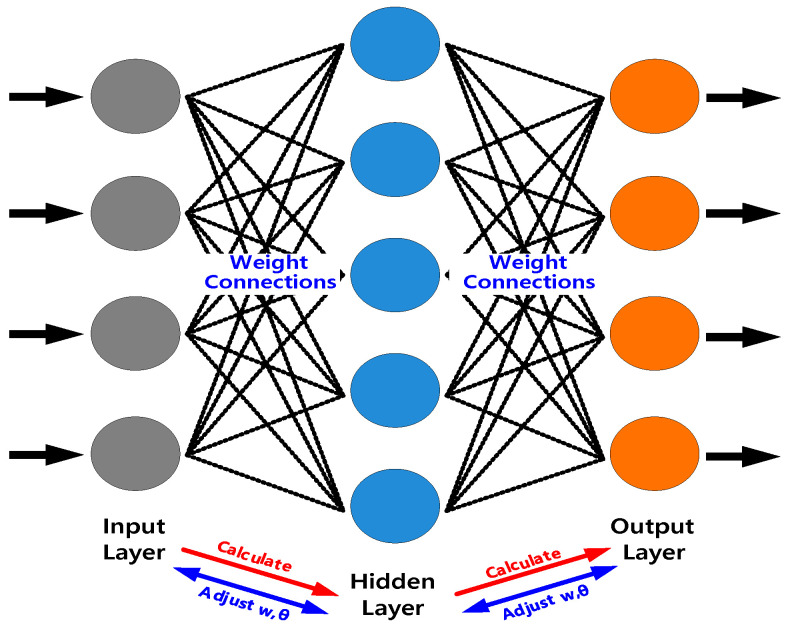
Artificial neural network (ANN) optimization process.

**Figure 8:**
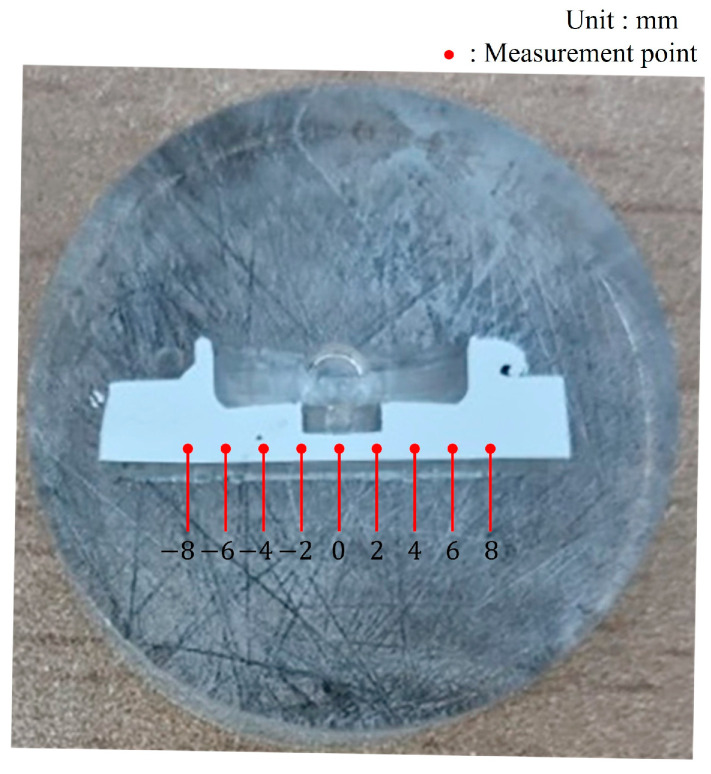
Measurement points for weld zone.

An ANN represents brain performance by simulating the structures of human synaptic connections. A feed-forward ANN known as a multilayer perceptron (MLP) employs several layers consisting of computational neurons. An MLP has connections between adjacent layers, with each neuron in a layer connected only to other neurons in the next layer. The first layer of the MLP computes the values of the input variables, whereas the hidden layer computes intermediate values with each weighted connection between the neurons. Finally, the last layer computes the output of the MLP. MLPs are trained by modifying these weights to target a computed output that achieves the desired prediction. Figure 7 is a diagram showing the optimization process. In this study, RS, DT, and PD were selected as the input data because they significantly affect the results of the FSSW process. The bonding strength and the hardness were selected as output data because they are the most important factors in the welding quality. As shown in Figure 8, the hardness was measured at every 2 mm from the center of the weld after cutting the specimen produced by FSSW to ensure the weld was visible. As the hardness of aluminum changes with heat, the specimens were manufactured using cold mounting. In addition, the hardness measurements were performed according to the ASTM-E384 standard and confirmed by pressing a square pyramid of diamond with a face angle of 136° using a Vickers hardness tester. The lowest hardness among the measured results was used.

## 3. Results and Discussion

### 3.1. Experiments

**Figure 9:**
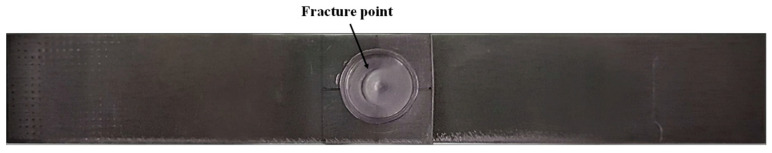
Fractured specimen after lap-shear test.

In the experiments, the bonding was examined according to the process parameters, with the bonding strength checked to determine if the bonding was successful. The bonding strength was measured by a lap-shear test using a universal testing machine as mentioned in Section 2.1. Figure 9 shows the fractured specimen after the lap-shear test.

### 3.2. FEA

**Figure 10:**
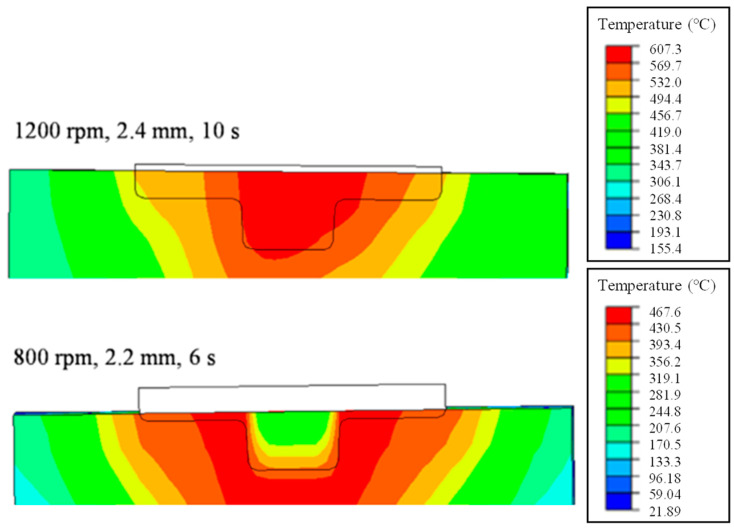
Typical cases of temperature distribution after the FSSW process.

FEA was performed according to the process parameters shown in Table 2. As the results from the FEA could not confirm whether the bonding occurred, the contact pressure and flow stress of the material at the given temperature, strain, and strain rate conditions and the velocity of the specified node were extracted and substituted into the bonding criterion. Figure 10 shows the results from the FEA of the FSSW process using the CEL technique in the ABAQUS software at RSs of 1200 rpm and 800 rpm, PDs of 2.4 mm and 2.2 mm, and DTs of 10 s and 6 s. In the case of an RS of 1200 rpm, PD of 2.4 mm, and DT of 10 s, sufficient frictional heat is generated, with the upper and lower sheets bonded. In contrast, in the case of an RS of 800 rpm, PD of 2.2 mm, and DT of 6 s, sufficient frictional heat is not generated; thus, the upper and lower sheets are not bonded.

### 3.3. Bonding Criterion

**Figure 11:**
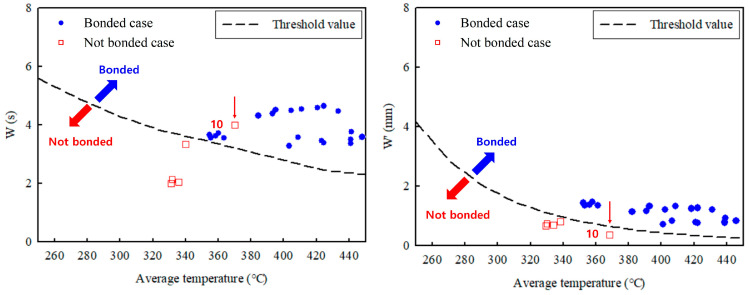
Results of bonding criterion according to threshold value.

The results obtained from the FEA were applied to the W and $W^{\prime }$ parameters. Based on the obtained values of W and $W^{\prime }$, if the threshold value was exceeded, it was judged that the bonding had occurred, and vice versa [21]. The average temperature values calculated during a time interval with non-zero pressure values correspond to the temperature at each point. The results are shown in Table 4 and compared with the actual test results. Figure 11 shows the values of W and $W^{\prime }$ according to temperature as graphs. When RS increases, hardness decreases depending on the position, owing to the local temperature increase in the weld zone [23].

The results for W and $W^{\prime }$ differ for condition No. 10. The W parameter indicates that bonding is possible, whereas the $W^{\prime }$ parameter indicates that bonding is not possible (as shown in the experimental results). These results indicate that the $W^{\prime }$ parameter is more suitable for the FSSW process because of the rapid velocity at the surface in the FSSW process; thus, the $W^{\prime }$ parameter with a velocity term seems more accurate. In contrast, a prior study showed that, for an FSW process, the W parameter could predict weldability [24,25]. Thus, the W parameter that does not consider the welding speed is more suitable for FSW compared to FSSW because the contact time of the tool is shorter and the welding speed is slower.

### 3.4. ANN

ANNs were trained for each objective function using observations for training (70%), testing (15%), and validation (15%) datasets to find the best combination of the prescribed parameter settings. The optimal combination was determined based on the minimum standard deviations of the residuals while testing different parameter combinations. Table 5 lists the ANN training parameters, with the neuron and layer count selected to ensure accurate predictions while avoiding both underfitting and overfitting.

**Figure 12:**
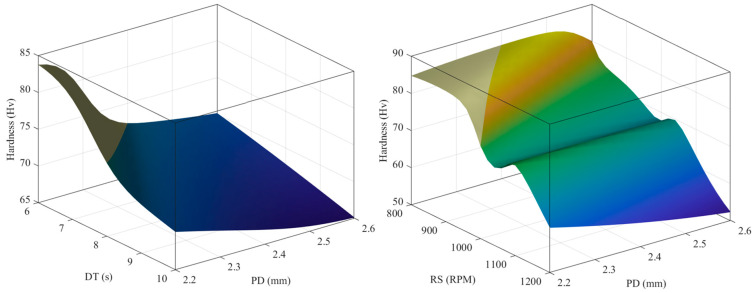
Predicted surface to hardness target based on design parameters.

**Figure 13:**
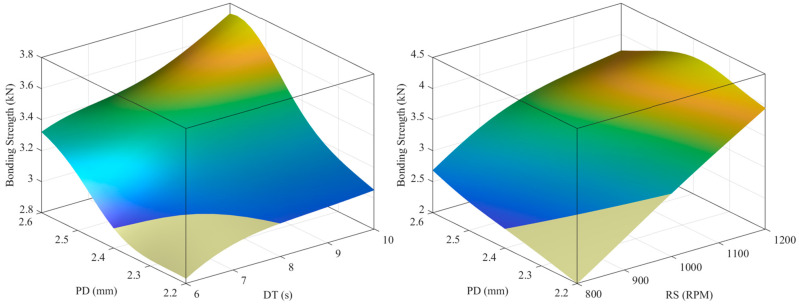
Predicted surface to bonding strength target based on design parameters.

Figure 12 and Figure 13 show the expected surface hardness and bonding strength values as functions of the process parameters, respectively. As shown in Figure 12, hardness decreases as DT, PD, and RS increase. In Figure 13, as PD, DT, and RS increase, the bonding strength increases.

Statistical criteria were also used to evaluate and compare the predictive abilities of the models, as shown in Table 6.

**Figure 14:**
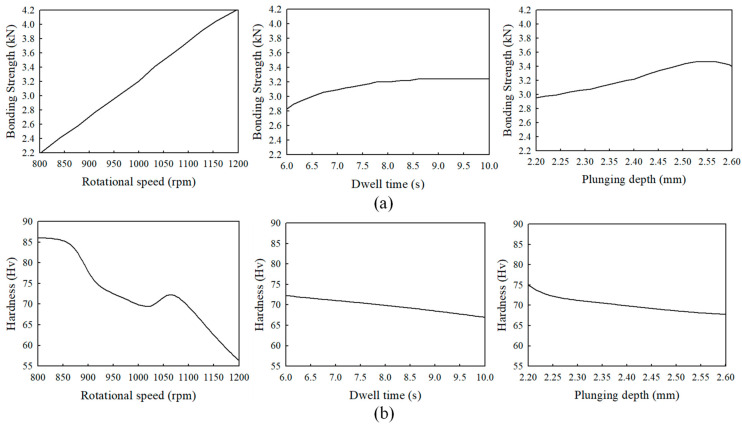
Variation of the objective functions (**a**) bonding strength and (**b**) hardness for each parameter when the other process parameters are held fixed.

Figure 14a shows the change in the bonding strength for each parameter when the other process parameters are held fixed, and Figure 14b shows the change in hardness. As shown in Figure 14a,b, when RS is 800 rpm, the bonding strength is predicted as 2.31 kN and the hardness as 86.02 Hv. When RS is 1200 rpm, the bonding strength is predicted as 4.2 kN and hardness as 56.4 Hv. In contrast, when the DT is 6 s, the bonding strength is predicted as 2.93 kN and the hardness as 72.30 Hv; when the PD is 2.2 mm, the bonding strength is predicted as 3.01 kN and the hardness as 74.97 Hv. As seen in the graph of Figure 14, RS significantly affects bonding strength and hardness (i.e., the slope of the RS graph is much greater than those of the DT and PD graphs).

**Figure 15:**
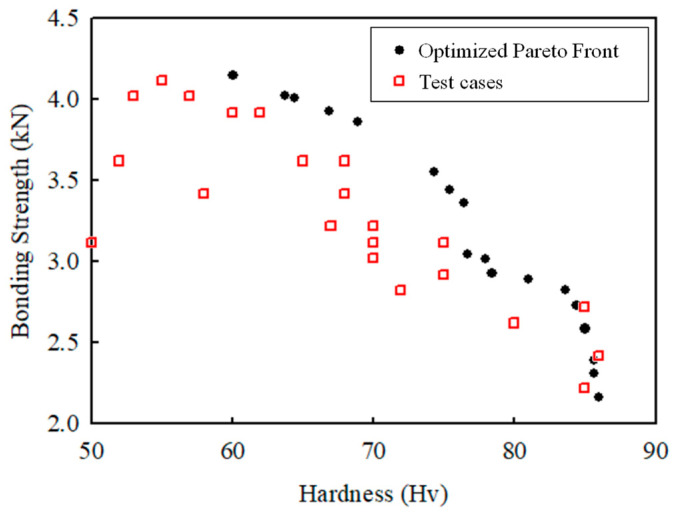
Results for the optimized Pareto front and all test cases.

Pareto fronts were obtained for each model using the “Non-Dominated Sorting Genetic Algorithm II” [26]. Figure 15 shows the achieved Pareto front for all cases in Table 2. All points can be selected, considering the design priorities. In this study, the first value of the Pareto front (the value with the largest bonding strength) was selected and compared with the actual experimental results, as shown in Table 7. Both the predicted and experimental results show similar values in terms of bonding strength and hardness. In previous research, Jo et al. optimized the FSSW process parameters using the Taguchi method [16], with only bonding strength set as the objective function; thus, the optimal process parameters were set as RS of 1400 rpm and DT of 8 s (PDs were not compared because the sheet thicknesses were different). In this study, as the objective functions are bonding strength and hardness, the results considering both are considered more useful. If only the bonding strength is used as the objective function, RS and DT are higher than necessary and hardness is lowered, resulting in a poor welding quality [27].

In future work, using these results, it will be possible to easily determine the bonding strength and hardness of the FSSW process. Furthermore, weldability can be predicted by using these results for processes similar to friction stir spot welding, such as refilled friction stir spot welding (RFSSW) and two-stage refilled friction stir spot welding (TFSSW), which are processes that have been recently studied.

## 4. Conclusions

In this study, an FSSW process using AA6061-T6 with 1.6-mm thickness was compared and analyzed using experiments, FEA, bonding criteria, and ANNs to optimize the bonding and process parameters. Based on the analysis and experiments, the following conclusions were drawn.

1. In the FSSW process, the pressure-time-flow criterion is more appropriate than the pressure-time criterion.
2. In the FSSW process using the pressure-time-flow criterion, it is possible to know whether bonding has occurred without a separate preliminary experiment.
3. Among the FSSW process parameters (RS, PD, DT), RS has the greatest effect on both the bonding strength and hardness.
4. When process parameters were optimized using ANN, the values were 1178.276 rpm for RS, 7.490 s for DT, and 2.402 mm for PD. The experimental value of the bonding strength was 4.0 kN and the predicted value was 4.147 kN, resulting in an error of 3.675%, while the hardness had an error of 3.197% (with an experimental value of 62 Hv and predicted value of 60.018 Hv).

## Figures and Tables

**Table 1:** Mechanical properties of the AA6061-T6 sheet.

Mechanical Property	Value
Young’s modulus	68.9 GPa
Poisson’s ratio	0.33
Tensile yield stress	276 MPa
Ultimate tensile strength	310 MPa
Vickers hardness	107 Hv

**Table 2:** Friction stir spot welding (FSSW) process parameters.

Tool Rotational Speed (rpm)	Dwell Time (s)	Plunging Depth (mm)
800	6	2.2
1000	8	2.4
1200	10	2.6

**Table 3:** Parameters for finite element (FE) model of AA6061-T6.

Parameters		Value	Reference
Young’s modulus (GPa)		69	[16]
Poisson’s ratio (*ν*)		0.33	[16]
Thermal expansion (1/°C)		2.8 × 10^−5^	[16]
Thermal conductivity (W/m·K)		1180 (at 20 °C) 225 (at 600 °C)	[16]
Friction coefficient (*μ*)		0.06 (600 °C)	[19]
		0.3 (100 °C)	[20]
Convective heat transfer coef., *h*_conv_ (W/m^2^·K)		20	[16]
Interface heat transfer coef., *h_inter_* (W/m^2^·K)		3500 (at pressure 2 MPa) 8000 (at pressure 8 MPa)	[16]
Johnson–Cook parameters	A	324.1	[18]
	B	113.8	
	C	0.002	
	m	1.34	
	n	0.42	
	D_1_	−0.77	
	D_2_	1.45	
	D_3_	−0.47	
	D_4_	0.0	
	D_5_	1.6	
Specific heat (J/kg·°C)		925 (at 20 °C) 1233 (at 600 °C)	[16]

**Table 4:** Bonding criteria, bonding strength, and hardness results in all cases.

No.	RS (RPM)	DT (s)	PD (mm)	W Parameter (s)	$W^{\prime }$ Parameter (mm)	Hardness (Hv)	Bonding Strength (kN)	Average Temperature (°C)
1	800	6	2.2	2.34	0.94	X	X	337.8
2	800	6	2.4	2.48	1.00	X	X	338.4
3	800	6	2.6	2.39	0.96	X	X	342.5
4	800	8	2.2	3.68	1.07	X	X	346.7
5	800	8	2.4	3.89	1.62	85	2.3	357.8
6	800	8	2.6	3.83	1.59	80	2.7	352.3
7	800	10	2.2	3.72	1.51	86	2.5	361.2
8	800	10	2.4	3.79	1.54	85	2.8	356.2
9	800	10	2.6	3.73	1.51	75	3.2	353.3
10	1000	6	2.2	4.35	0.62	X	X	376.8
11	1000	6	2.4	4.56	1.32	72	2.9	391.0
12	1000	6	2.6	4.49	1.30	70	3.3	382.2
13	1000	8	2.2	4.67	1.37	75	3.0	402.3
14	1000	8	2.4	4.82	1.43	70	3.2	422.1
15	1000	8	2.6	4.76	1.40	68	3.5	418.3
16	1000	10	2.2	4.64	1.36	70	3.1	431.1
17	1000	10	2.4	4.71	1.48	67	3.3	408.6
18	1000	10	2.6	4.69	1.48	65	3.7	392.8
19	1200	6	2.2	3.45	0.88	68	3.7	401.1
20	1200	6	2.4	3.74	1.00	60	4.0	406.7
21	1200	6	2.6	3.55	0.92	57	4.1	422.2
22	1200	8	2.2	3.68	0.97	62	4.0	438.7
23	1200	8	2.4	3.94	1.08	55	4.2	439.2
24	1200	8	2.6	3.75	1.00	52	3.7	445.6
25	1200	10	2.2	3.54	0.92	58	3.5	438.8
26	1200	10	2.4	3.76	1.00	53	4.1	445.8
27	1200	10	2.6	3.62	0.95	50	3.2	421.0

**Table 5:** Parameters of trained artificial neural networks (ANNs).

Objective	Hardness	Bonding Strength
Neurons in the input layer	3	3
Number of hidden layers	1	1
Neurons in the hidden layer	4	4
Neurons in the out layer	1	1
Training algorithm	Levenberg–Marquardt Back-Propagation	Levenberg–Marquardt Back-Propagation
Activation function (Hidden layer)	Tansig	Tansig
Activation function (Output layer)	Purelin	Purelin
Validation data fraction (%)	15	15
Test data fraction (%)	15	15

**Table 6:** Statistical characteristics of the surrogate model.

Objective	Mean Square Error (MSE)	Root Mean Square Error (RMSE)	R
Hardness	1.159929	1.077	0.997
Bonding Strength	0.005329	0.073	0.997

**Table 7:** Overall optimization summary.

Parameter/Objective	ANN Optimum
RS (rpm)	1178.276
DT (s)	7.490
PD (mm)	2.402
Hardness (Predicted)	60.018
Hardness (Experiment)	62.000
Bonding strength (Predicted)	4.147
Bonding strength (Experiment)	4.000
Relative error (Hardness)	3.197%
Relative error (Bonding strength)	3.675%

## Data Availability

The data presented in this study are available on request from the corresponding author.
